# Novel Indole-Based Analogs of Melatonin: Synthesis and *in Vitro* Antioxidant Activity Studies

**DOI:** 10.3390/molecules15042187

**Published:** 2010-03-29

**Authors:** Hanif Shirinzadeh, Burcu Eren, Hande Gurer-Orhan, Sibel Suzen, Seçkin Özden

**Affiliations:** 1 Department of Pharmaceutical Chemistry, Faculty of Pharmacy, Ankara University, 06100 Tandogan, Ankara, Turkey; 2 Department of Pharmaceutical Toxicology, Faculty of Pharmacy, Ege University, 35100 Bornova, Izmir, Turkey

**Keywords:** indole, hydrazone, hydrazide, melatonin, synthesis, antioxidant activity

## Abstract

The aim of this study was to synthesize and examine possible *in vitro* antioxidant effects of indole-based melatonin analogue compounds. As a part of our ongoing study nineteen indole hydrazide/hydrazone derivatives were synthesized, characterized and their *in vitro* antioxidant activity was investigated by three different assays: by evaluating their reducing effect against oxidation of a redox sensitive fluorescent probe, by examining their protective effect against H_2_O_2_-induced membrane lipid peroxidation and by determining their inhibitory effect on AAPH–induced hemolysis of human erythrocytes. The results indicated significant strong antioxidant activity for most of the compounds, when compared to melatonin.

## 1. Introduction

Antioxidants are molecules which can interact with free radicals and stop their chain reactions before important and essential molecules are damaged. As oxidative stress is an important part of many diseases, the use of antioxidants is intensively studied in medicinal chemistry, particularly as treatments for vital diseases such as stroke, cancer and neurodegenerative diseases. Free radicals are produced basically during cellular metabolism and some functional activities and have essential roles in cell signaling, apoptosis and gene expression. On the other hand, excessive free radical attack can damage DNA, proteins and lipids, resulting very important diseases. Antioxidants can decrease the oxidative damage by reacting with free radicals or by inhibiting their activity.

It is well known that indole derivatives, extensively present in natural compounds, are very important substances for their medicinal and biological aspects. Antioxidant effects of the indole ring- containing substance melatonin (MLT) have been well described and evaluated by Tan *et al.* [1]. It is a highly conserved molecule that it acts as a free radical scavenger and a broad-spectrum antioxidant [2]. Studies also showed the role of MLT and its derivatives in many physiological processes and therapeutic functions, such as the regulation of circadian rhythm and immune functions [3,4,5,6]. Melatonin is known to be a potent *in vitro* antioxidant as well as powerful *in vivo* radical scavenger. In *in vitro* conditions melatonin exhibited potent antioxidant activity in a linoleic acid emulsion system. It also showed potent superoxide radical scavenging activity, higher than either quercetin or BHT [7]. 

Recent research has proved that the indole ring in the MLT molecule is the reactive center dealing with oxidants due to its high resonance stability and very low activation energy barrier towards free radical reactions [8,9,10]. Indole is found to reduce cisplatin-induced reactive oxygen species formation [11] and scavenge hydroxyl radical directly [12]. It was shown that alkyl-substituted indole derivatives can trap ABTS+• and DPPH indicating that the alkyl group attached to indole is of importance for the antioxidant activity [13]. The hydrogen peroxide scavenging activity of melatonin showed that the scavenging activity augmented with increasing concentrations of melatonin. This result may be illustrative of melatonin's ability to inhibit lipid peroxidation [14]. A series of dihydroindenoindole derivatives containing methoxy, halogen, and hydroxyl groups was synthesized and showed effective inhibition against DPPH·, ABTS·^+^, DMPD·^+^, and superoxide anion radicals compared to standard antioxidants [15]. 2-Phenylindole derivatives significantly inhibited lipid peroxidation at 10^-3 ^M concentration [16]. Indole-3-propionamide derivatives also exhibited important antioxidant activity compared to melatonin [17]. A large variety of synthetic compounds have been identified as potent *in vitro* antioxidants, yet many of these compounds have not provided great clinical benefits, and some produced side effects. In our earlier study [18] two sets of indole derivatives, with changes in the 5-methoxy and 2-acylaminoethyl groups of MLT were synthesized and tested for their *in vitro* antioxidant potency in the DPPH, superoxide dismutase and lipid peroxidation (LP) assays. With a few exceptions most of the compounds tested showed significant antioxidant activity at concentrations comparable with or much higher than that of MLT. These results prompted us to synthesize more indol-3-aldehyde hydrazone and hydrazide derivatives.

As a part of our ongoing study nineteen indole-based MLT analogue hydrazide/hydrazone derivatives were now synthesized and their antioxidant activity was investigated *in vitro* by three different assays: by evaluating their reducing effect against oxidation of a redox sensitive fluorescent probe, DCFH-DA, by investigating their protective effect against H_2_O_2_-induced membrane lipid peroxidation and by determining their inhibitory effect on 2,2’-azobis(2-amidinopropane hydrochloride) (AAPH) –induced hemolysis of human erythrocytes. Human erythrocytes were chosen as a biological model because they are readily available cells that are sensitive to oxidative damage. The results were compared with MLT. All the indole-based MLT analogue compounds except those previously synthesized (**1a** [19,20], **1j** [21] and **1r** [22]) were characterized on the basis of ^1^H- and ^13^C-NMR, mass and FT-IR spectra and elemental analysis.

## 2. Results and Discussion

**Figure 1:**
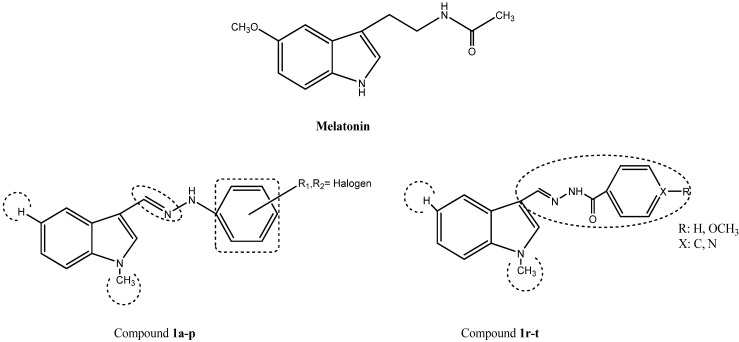
Parts of the MLT molecule modified to develop new indole-based MLT analogue compounds.

The present work aimed to synthesize, characterize and investigate the potential antioxidant effects of indole-based MLT analogue hydrazide/hydrazone derivatives by several *in vitro* test models. Based on MLT, *N*-acetyl-5-methoxytryptamine, a well-known antioxidant, free radical scavenger, and neuroprotectant, new indole imines were developed. Three parts of the MLT molecule were modified to develop new indole-based MLT analogue compounds. These modifications were done mainly on the acylamino group (Figure 1). 

**Scheme 1:**
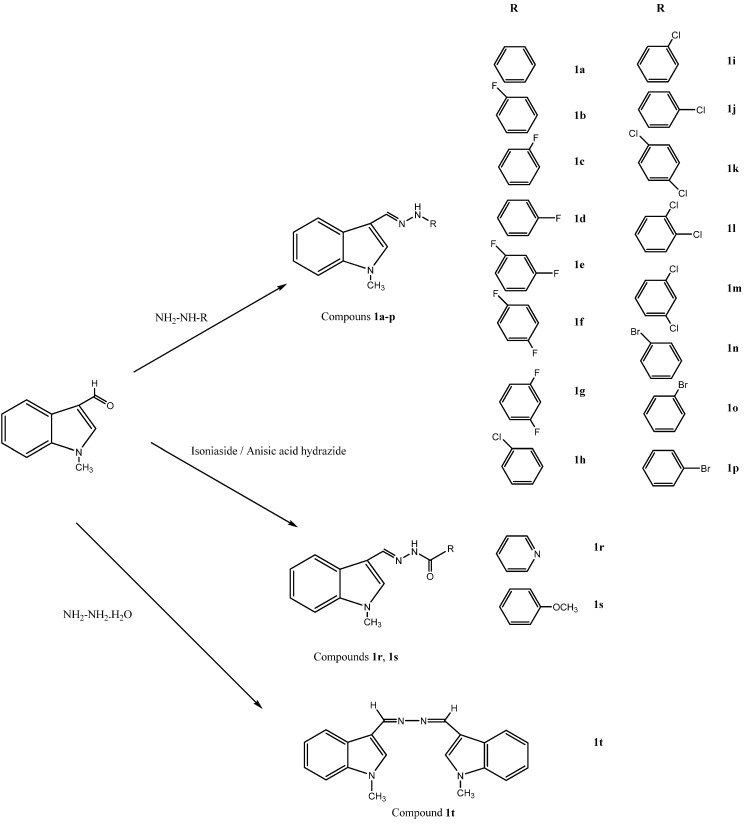
Synthetic route to indole-based melatonin analogue hydrazide/hydrazone derivatives.

These chemically significant modulations of the lead structure were made at three different points: the methoxy group replaced with hydrogen at the 5-position of the indole ring (modification I) and acetylaminoethyl side chain modified by formation of imine (modification II) and hydrogen replaced with methyl on nitrogen (modification III). Particular attention was dedicated to the role of the 5-methoxy group, which was eliminated. These modifications resulted in a set of compounds having different physical properties, lipophilicity and different substitution patterns on the indole nucleus. This helped to investigate the effect of substituents with different electronic and lipophilic properties on the antioxidant activity of new indole derivatives (Scheme 1). 

### 2.1. Effects of synthesized indole derivatives on cellular ROS

**Figure 2:**
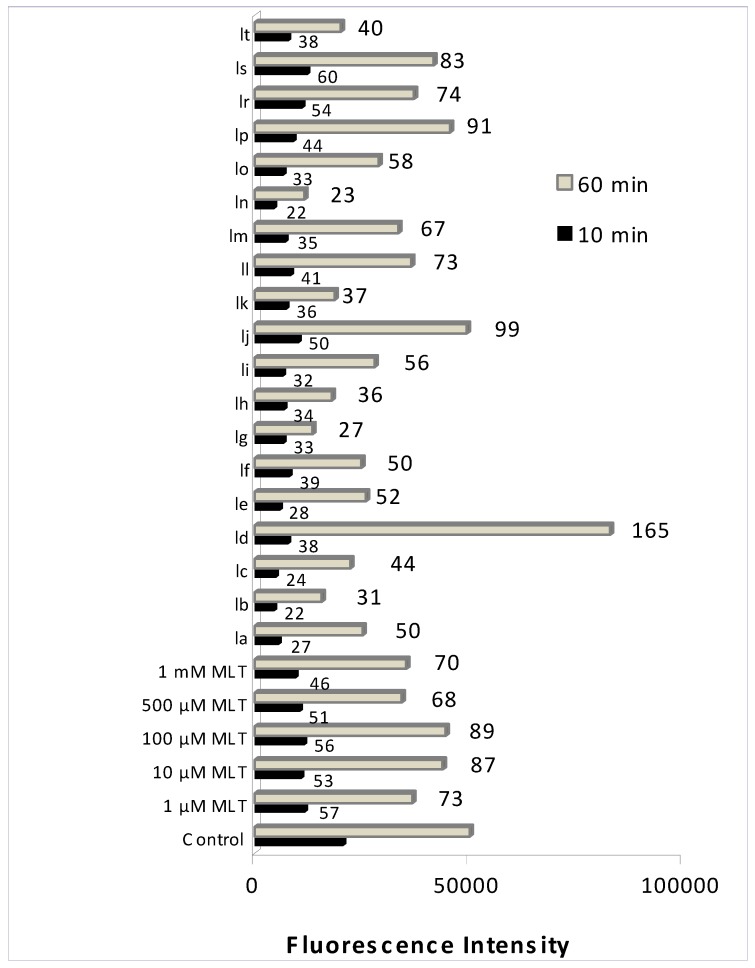
Oxidation of DCFH *via* ROS in erythrocytes after the incubation with various concentrations of MLT or 10 µM synthesised indole derivatives for 10 and 60 min. Values are mean ±SD of three individual experiments. Values above the bars are % control values.

The protective effect of newly synthesized indole-based MLT analogue against DCFH-DA oxidation was determined in human erythrocytes that were preloaded with the fluorescent probe. In cells, DCFH-DA locates in the cytosol and reflects cellular ROS formation. Oxidation of the probe was screened at various time intervals up to 60 min. At 10 min incubation all the synthesized indole derivatives except **1r** and **1s** were found to have potent antioxidant activity, even higher than MLT itself. Among those synthesized indoles with changes in the aromatic side chain, *p*-halogenations were found to decrease antioxidant activity compared to *o*- and *m*- substitution of the same halogen atom (Figure 2). A significant further decrease in the antioxidant activity of *p*-halogenated compounds was observed at 60 min incubation indicating a possible oxido-reductive reaction took place in incubations with those derivates or a possible structural change in those analogues resulting in loss of their antioxidant effect (Figure 2). On the other hand *m*-halogenations in the aromatic side chain were observed to eliminate the time dependency of the antioxidant effectiveness. This can be seen from unchanged DCFH oxidation with **1b**, **1h**, **1k** and **1n** at 10 min and 60 min incubations. **1r** and **1s** had the weakest antioxidant activity (almost equal to MLT) among all tested compounds. This can be explained by their structural difference from the rest of the synthesized compounds, mainly by having no aromatic halogenations and having a carbonyl group on the side chain. The results also indicate a biphasic pattern in the antioxidant effect of MLT; higher effect in lower concentrations (1 µM) and higher concentrations (500–1000 µM) whereas lower antioxidant effect was observed in between (10, 100 µM) (Figure 2).

### 2.2. Inhibitory effect of synthesized indole derivatives on hydrogen peroxide-induced peroxidation of human erythrocyte membranes

**Figure 3:**
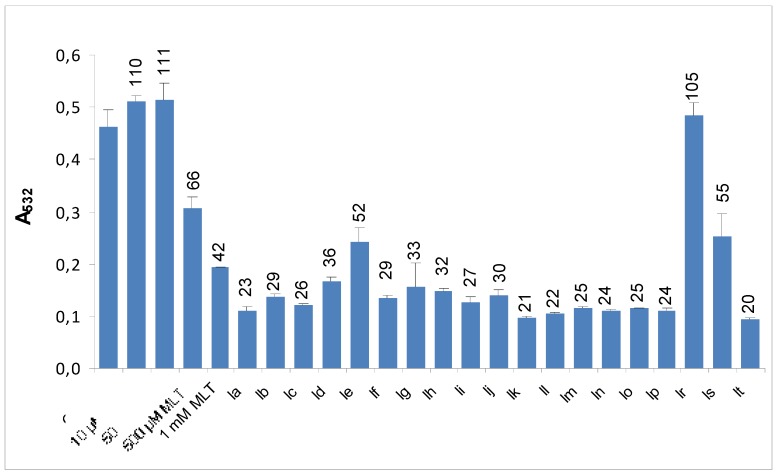
Effects of various concentrations of MLT and 10 μM synthesised indole derivatives on H_2_O_2_-induced lipid peroxidation in erythrocyte membranes. MDA values were determined as an endproduct of lipid peroxidation. Values are mean ±SD of three individual experiments. Values above the bars are % control values.

Once ROS are formed, one of the subsequent detrimental outcomes is peroxidation and oxidative destruction of polyunsaturated fatty acids (PUFA) in cell membranes. The process is initiated by an oxidizing radical that is capable of abstracting one hydrogen atom from PUFA. After several rearrangement and oxidation reactions, generated lipid hydroperoxides decompose to a wide range of products, mainly small molecule alkanes and aldehydes. Among those aldehydes, malondialdehyde (MDA), assayed by the thiobarbituric acid (TBA) assay, is the most widely used biomarker of oxidative damage to lipids. In the present study we investigated protective effect of synthesized indole derivatives on H_2_O_2_-induced MDA formation in erythrocyte membranes. Figure 3 shows the inhibitory effect of MLT and the synthesized indole derivatives on H_2_O_2_-induced peroxidation of human erythrocytes *in vitro*. 

**Figure 4:**
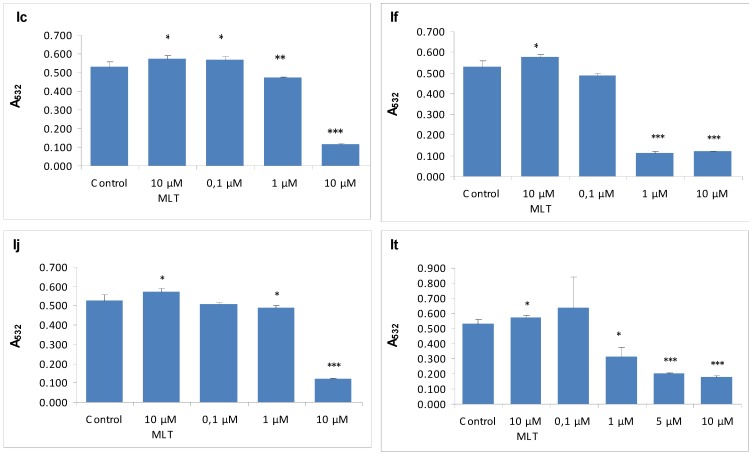
Effects of various concentrations of **1c**, **1f**, **1j** and **1t** on H_2_O_2_-induced LP. Values are mean ±SD of three individual experiments. * p < 0.05, ** p < 0.01, *** p < 0.005 significantly different from control.

MLT, a well known antioxidant, was used as a reference control for comparison purposes. The results obtained in this model were in accordance with data from ROS-mediated DCFH oxidation assay; all analogues except **1r** and **1s** were effective in protecting erythrocyte membranes from the attack of H_2_O_2_ and further lipid peroxidation (Figure 3). By combining those findings lack of protective effect of **1r** and **1s** in H_2_O_2_-induced erythrocyte membrane LP might be explained by the lack of their ROS scavenging ability. Furthermore we did additional experiments with four of the selected synthesised indole derivatives (**1c**, **1f**, **1j** and **1t**) in order to see the concentration dependency of their protective effect against H_2_O_2_-induced erythrocyte membrane LP. As presented in Figure 4 several of the compounds that were found to have higher protective effect against H_2_O_2_-induced erythrocyte membrane LP than MLT at same concentration (10 μM) were found to have same effect even at their lower concentrations (Figure 4 - **1f**, **1t**). These finding indicate that several of the newly synthesized indole derivatives may exert their antioxidant effect even at lower concentrations which make them promising candidates as antioxidant drugs. 

### 2.3. The antioxidant effect of synthesized indole derivatives on AAPH-induced oxidative hemolysis

The hemolysis of erythrocytes induced by free radicals is a good model system to study both oxidative damage and protection by antioxidants. Thermal decomposition of AAPH results in free radicals which attack the erythrocyte membranes to induce LP [23]. Once LP chain reaction starts the RBC membranes are quickly damaged, leading to hemolysis. On the other hand if antioxidants are added to red blood cells (RBCs) they would react with the radicals and inhibit hemolysis. Hemolysis does not start at the beginning of the reaction because the endogenous antioxidants of erythrocytes protect the membrane against oxidative damage induced by AAPH. Hemolysis takes place after the endogenous antioxidants are depleted thoroughly, generating an inhibition period (t_lag_) [23]. Oxidative hemolysis of the erythrocytes was screened for 5 h. As can be seen in Table 1, presenting the quantitative indice (t_lag_) obtained from this assay, MLT was found to have highest antioxidant activity among all models tested in the present study. This protective effect of MLT against AAPH-induced oxidative hemolysis indicates free radical scavenging effect of MLT which was previously shown by Zhao *et al.* [24]. All tested MLT analogues in this model was found to have either equal or higher t_lag_ values compared to MLT. Similar to the findings from other models that we used **1s** was found to have the least antioxidant effect where **1r** did not exert any radical scavenging effect at all. These findings may suggest that aromatic halogenation increases the free radical scavenging and therefore antioxidant effect of indole derivatives.

**Table 1:** Quantitative indicator measured from AAPH-induced erythrocyte hemolytic curves. Values are the means of data obtained from three separate curves. Data represent mean of three different curves for each compound. tlag; lag time before the starting of hemolysis.

Substrate	t_lag_ (min)
**AAPH**	97
**10 μM MLT**	128
**1 MM MLT**	>300
**10 μM 1a**	160
**10 μM 1b**	137
**10 μM 1d**	160
**10 μM 1g**	160
**10 μM 1j**	160
**10 μM 1k**	160
**10 μM 1l**	160
**10 μM 1n**	131
**10 μM 1p**	160
**10 μM 1r**	97
**10 μM 1s**	124
**10 μM 1t**	124

Like other indole derivatives and tryptophan metabolites, MLT has inherent redox properties due to the presence of an electron-rich aromatic ring system, which allows the indoleamine to easily function as an electron donor [25]. A number of oxygen-centered radicals and other reactive species have been shown to be capable of oxidizing MLT in various experimental systems. It is possible that making the indole ring more stable electronically helped to act as a better electron donor. Introduction of an imine group in to the side chain increased the stability of the indole molecule by helping the delocalization of the electrons. This might help to have high free radical scavenging activity. Also according to Reiter [26] MLT scavenges the radicals most likely *via* electron donation, thereby neutralizing the radicals and generating nitrogen centered radical, the indolyl (or melatonyl) cation radical. These results suggest a new approach for the *in vitro* antioxidant activity properties and structure activity relationship of 1 and 3 substituted indole ring regarding to antioxidant activity. 

## 3. Experimental

### 3.1. Material and methods

Uncorrected melting points were determined with a Büchi SMP-20 apparatus. The ^1^H- and ^13^C- NMR spectra were measured with a Varian 400 MHz instrument using TMS internal standard and DMSO-d_6 _as solvent. ESI Mass spectra were determined on a Waters Micromass ZQ. FT-IR spectra were recorded on a Jasco 420 Fourier Transform apparatus. Elemental analyses were performed using a CHNS-932 instrument (LECO). All spectral analysis was performed at the Central Laboratory of the Faculty of Pharmacy, Ankara University. Chromatography was carried out using Merck silica gel 60 (230–400 mesh ASTM). The chemical reagents used in synthesis were purchased from Sigma (Germany) and Aldrich (USA).

### 3.2. Chemistry

The target imines were derived from 1-methyl-1*H*-indole-3-carboxaldehyde and appropriate hydrazine or hydrazide derivatives using simple reaction strategies. For the synthesis of compounds **1a–p** a methodology similar to that of Kidwai *et al.* [27] has been adopted. The hydrazones **1r** and **1s** were also prepared from the reaction of equimolar amounts of hydrazide with 1-methyl-1*H*-indole-3-carboxaldehyde in the presence of ethanol. Finally *N,N^’^*-bis-(1-methylindole-3-ylmethylene)-hydrazine derivatives were synthesized using hydrazine hydrate with 1-methyl-1*H*-indole-3-carboxaldehyde in the presence of ethanol. All the new compounds (except **1a** [19,20], **1j** [21] and **1r** [22]) were characterized on the basis of their spectral and analytical data.

### 3.3. General procedure for the synthesis of compounds ***1a–p***

1-Methyl-1*H*-indole-3-carboxaldehyde (1 mmol) and phenyl hydrazine or its derivatives (1.3 mmol) in EtOH (10 mL) was heated for 30 min on the hot water bath in the presence of CH_3_COONa (0.4 g). On cooling, the precipitate was collected washed with cold EtOH and recristallized from EtOH to give **1a–p** with 44 to 82% yield.

*1-Methylindole-3-carboxaldehyde (2-fluorophenyl)hydrazone* (**1b**). Yield 65.2%, m.p. 130–131 ºC; ^1^H-NMR: δ 3.78 (3H,s), 6.76 (1H, m), 7.09 (2H, m), 7.24 (2H, m), 7.46 (2H, m), 7.62 (1H, s, azomethine-CH), 8.23 (1H, d), 8.31 (1H, s) 9.72 (1H, s, hydrazine-NH); ^13^C-NMR: δ 33.11, 110.74, 112.30, 113.78, 115.37, 115.54, 117.90, 121.03, 122.42, 123.09, 125.23, 125.73, 132.38, 135.00, 137.97, 148.39 (azomethine-C), 150.76; ESI MS m/z 268 (M+1, %100), 269 (M+2); Anal. Calcd. for C_16_H_14_N_3_F: C, 71.89%; H, 5.28%; N, 15.72%. Found: C, 71.35%; H, 4.48%; N, 15.76%. FT-IR (KBr) cm^-1 ^1580 (C=N, azomethine stretch), 3295 (N-H stretch).

*1-Methylindole-3-carboxaldehyde (3-fluorophenyl)hydrazone* (**1c**). Yield 49%, m.p. 144–145 ºC; ^1^H-NMR: δ 3.80 (3H,s), 6.46 (1H, m), 6.80 (2H, m), 7.24 (3H, m), 7.48 (1H, d), 7.66 (1H, s), 8.11 (1H, s, azomethine-CH), 8.23 (1H, d), 10.16 (1H, s, hydrazine-NH); ^13^C-NMR: δ 33.22, 98.19, 104.27, 108.20, 110.76, 112.21, 121.04, 122.30, 123.09, 125.21, 131.35, 132.35, 136.25, 138.16, 148.78 (azomethine-C), 162.96, 165.35; ESI MS m/z 268 (M+1, %100), 269 (M+2); Anal. Calcd. for C_16_H_14_N_3_F: C, 71.89%; H, 5.27%; N, 15.72%. Found: C, 69.30%; H, 5.05%; N, 15.13%. FT-IR (KBr) cm^-1 ^1582 (C=N, azomethine stretch), 3318 (N-H stretch).

*1-Methylindole-3-carboxaldehyde (4-fluorophenyl)hydrazone* (**1d**). Yield 68.9%, m.p. 150–151 ºC; ^1^H-NMR: δ 3.80 (3H,s), 7.04 (4H, m), 7.23 (2H, m), 7.47 (1H, d), 7.61 (1H, s), 8.08 (1H, s, azomethine-CH), 8.24 (1H, d,), 9.83 (1H, s, hydrazine-NH); ^13^C-NMR: δ 33.28,110.68, 112.47, 112.88, 116.36, 120.88, 122.39, 123.03, 125.24, 131.86, 135.26, 138.14, 143.58 (azomethine-C), 154.75, 157.05: ESI MS m/z 268 (M+1, %100), 269 (M+2); Anal. Calcd. for C_16_H_14_N_3_F: C, 71.89%; H, 5.28%; N, 15.72%. Found: C, 71.31%; H, 5.24%; N, 15.19%. FT-IR (KBr) cm^-1 ^1570 (C=N, azomethine stretch), 3331 (N-H stretch).

*1-Methylindole-3-carboxaldehyde (2,4-difluorophenyl)hydrazone* (**1e**). Yield 68.8%, m.p. 137–138 ºC; ^1^H-NMR: δ 3.78 (3H,s), 7.02 (1H, m), 7.18 (3H, m), 7.45 (2H, m), 7.62 (1H, s), 8.22 (1H, d), 8.30 (1H, s, azomethine-CH), 9.66 (1H, s, hydrazine-NH); ESI MS m/z 286 (M+1, %100), 287 (M+2); Anal. Calcd. for C_16_H_13_N_3_F_2_: C, 67.36%; H, 4.59%; N, 14.73%. Found: C, 66.99%; H, 4.60%; N, 14.68%. FT-IR (KBr) cm^-1 ^1598 (C=N, azomethine stretch), 3340 (N-H stretch).

*1-Methylindole-3-carboxaldehyde (2,5-difluorophenyl)hydrazone* (**1f**). Yield 70.5%, m.p. 156–157 ºC; ^1^H-NMR: δ 3.78 (3H,s), 6.37 (1H, m), 6.59 (2H, dd), 7.17 (1H, dd), 7.22 (2H, m), 7.45 (1H, d), 7.67 (1H, s, azomethine-CH), 8.11 (1H, s), 8.17 (1H, d), 10.40 (1H, s, hydrazine-NH); ^13^C-NMR: δ 33.35, 92.55, 94.49, 110.82, 111.86, 121.21, 122.22, 123.16, 125.16, 132.93, 137.40, 138.17, 149.21 (azomethine-C), 162.93, 165.33; ESI MS m/z 286 (M+1, %100), 287 (M+2); Anal. Calcd. for C_16_H_13_N_3_F_2_:C, 67.36%; H, 4.59%; N, 14.73%. Found: C, 66.41%; H, 4.48%; N, 14.55%. FT-IR (KBr) cm^-1 ^1591 (C=N, azomethine stretch), 3433 (N-H stretch).

*1-Methylindole-3-carboxaldehyde (3,5-difluorophenyl)hydrazone* (**1g**). Yield 44.9%, m.p. 120–121 ºC; ^1^H-NMR: δ 3.81 (3H, s), 6.45 (1H, m), 7.15 (2H, m), 7.25 (2H, m), 7.49 (1H, d), 7.70 (1H, s, azomethine-CH), 8.18 (1H, d), 8.36 (1H, s), 10.03 (1H, s, hydrazine-NH); ESI MS m/z 286 (M+1, %100); Anal. Calcd. for C_16_H_13_N_3_F_2_: C, 67.36%; H, 4.59%; N, 14.72%. Found: C, 67.76%; H, 4.61%; N, 14.52%. FT-IR (KBr) cm^-1 ^1591 (C=N, azomethine stretch), 3332 (N-H stretch).

*1-Methylindole-3-carboxaldehyde (2-chlorophenyl)hydrazone* (**1h**). Yield 82.3%, m.p. 134–135 ºC; ^1^H-NMR: δ 3.82 (3H,s), 6.73 (1H, m), 7.20-7.32 (4H, m), 7.50 (2H, dd), 7.67 (1H, s, azomethine-CH), 8.24 (1H, d), 8.47 (1H, s) 9.43 (1H, s, hydrazine-NH); ^13^C-NMR: δ 33.35, 110.83, 112.17, 113.84, 116.24, 119.06, 121.14, 122.39, 123.15, 125.23, 128.83, 129.93, 132.72, 138.21, 138.94, 142.69 (azomethine-C), 150.76; ESI MS m/z 284 (M^+^, %100), 286 (M+2); Anal. Calcd. for C_16_H_13_N_3_Cl: C, 67.72%; H, 4.97%; N, 14.81%. Found: C, 67.75%; H, 4.36%; N, 14.09%. FT-IR (KBr) cm^-1 ^1592 (C=N, azomethine) stretch), 3328 (N-H stretch).

*1-Methylindole-3-carboxaldehyde (3-chlorophenyl)hydrazone* (**1i**). Yield 78.4%, m.p. 135–136 ºC; ^1^H- NMR: δ 3.78 (3H,s), 6.46 (1H, m), 6.55 (1H, dd, 6.92 (1H, dd), 7.00 (1H, m), 7.20 (3H, m), 7.47 (1H, d), 7.64 (1H, s), 8.08 (1H, s, azomethine-CH), 8.18 (1H, d), 10.08 (1H, s, hydrazine-NH); ^13^C-NMR: δ 33.34, 110.70, 111.19, 112.14, 117.50, 121.04, 122.20, 123.11, 125.21, 131.42, 132.42, 134.44, 136.49, 138.15, 148.13 (azomethine-C), 162.96, 165.35; ESI MS m/z 284 (M^+^, %100), 286 (M+2); Anal. Calcd. for C_16_H_14_N_3_Cl: C, 67.72%; H, 4.97%; N, 14.81%. Found: C, 67.57%; H, 4.81%; N, 14.77%. FT-IR (KBr) cm^-1 ^1592 (C=N, azomethine stretch), 3302 (N-H stretch).

*1-Methylindole-3-carboxaldehyde (2,5-dichlorophenyl)hydrazone* (**1k**). Yield 78.3%, m.p. 150–151 ºC; ^1^H-NMR: δ 3.83 (3H,s), 6.76 (1H, dd), 7.26 (3H, m), 7.34 (1H, d), 7.45 (1H, d), 7.52 (1H, d), 7.74 (1H, s, azomethine-CH), 8.14 (1H, d), 8.52 (1H, s), 9.68 (1H, s, hydrazine-NH); ^13^C-NMR: δ 33.41, 111.01, 111.76, 112.80, 114.83, 118.23, 121.30, 121.94, 123.24, 125.20, 131.36, 133.28, 138.23, 140.37, 143.73 (azomethine-C), 162.93, 165.33; ESI MS m/z 318 (M^+^, %100), 320 (M+2), 322 (M+4); Anal. Calcd. for C_16_H_13_N_3_Cl_2_: C, 59.97%; H, 4.12%; N, 13.21%. Found: C, 59.97%; H, 4.18%; N, 13.23%. FT-IR (KBr) cm^-1 ^1592 (C=N, azomethine) stretch), 3315 (N-H stretch).

*1-Methylindole-3-carboxaldehyde (3,4-dichlorophenyl)hydrazone* (**1l**). Yield 89.3%, m.p. 160–161 ºC; ^1^H-NMR: δ 3.80 (3H, s), 6.95 (1H, dd), 7.15 (1H, d), 7.24 (2H, m), 7.42 (1H, d), 7.49 (1H, d), 7.68 (1H, s, azomethine-CH), 8.10 (1H, s), 8.18 (1H, d), 10.25 (1H, s, hydrazine-NH); ^13^C-NMR: δ 33.36, 110.85, 11.95, 112.30, 112.72, 118.56, 121.12, 122.18, 123.16, 125.16, 131.61, 132.13, 132.75, 137.18, 138.16, 146,70 (azomethine-C), 154,07, 156.38, 168.66; ESI MSm/z 318 (M^+^, %100), 320 (M+2); Anal. Calcd. for C_16_H_13_N_3_Cl_2_: C, 60.39%; H, 4.12%; N, 13.21%. Found: C, 60.16%; H, 4.16%; N, 12.96%. FT-IR (KBr) cm^-1 ^1593 C=N (azomethine) stretch band, 3433 N-H stretch band.

*1-Methylindole-3-carboxaldehyde (3,5-dichlorophenyl)hydrazone* (**1m**). Yield 80%, m.p. 161–162 ºC; ^1^H-NMR: δ 3.81 (3H, s), 6.77 (1H, t), 6.95 (2H, d), 7.25 (2H, m), 7.49 (1H, d), 7.72 (1H, s, azomethine-CH), 8.14 (2H, d and s), 10.35 (1H, s, hydrazine-NH); ^13^C-NMR: δ 34.41, 110.06, 110.94, 111.77, 116.75, 121.24, 122.04, 123.22, 125.17, 133.07, 135.31, 137.92, 138.19, 148,74 (azomethine-C); ESI MS m/z 318 (M^+^, %100), 320 (M+2); Anal. Calcd. for C_16_H_13_N_3_Cl_2_: C, 60.39%; H, 4.12%; N, 13.21%. Found: C, 60.26%; H, 3.80%; N, 13.18%. FT-IR (KBr) cm^-1 ^1586 (C=N, azomethine stretch), 3312 (N-H stretch).

*1-Methylindole-3-carboxaldehyde (2-bromophenyl)hydrazone* (**1n**). Yield 65.8%, m.p. 152–153 ºC; ^1^H-NMR: δ 3.82 (3H,s), 6.68 (1H, m), 7.23 (2H, m), 7.33 (1H, m), 7.53 (3H, m), 7.66 (1H, s, azomethine-CH), 8.23 (1H, d), 8.49 (1H, s) 9.15 (1H, s, hydrazine-NH); ^13^C-NMR: δ 33.33, 106.22, 110.80, 112.13, 114.32, 119.79, 121.12, 122.33, 123.13, 125.24, 129.34, 132.70, 133.12, 138.20, 139.68, 143.68 (azomethine-C), 150.76; ESI MS m/z 328 (M^+^, %100), 330 (M+2, %100), 331 (M+3); Anal. Calcd. for C_16_H_14_N_3_Br: C, 58.55%; H, 4.30%; N, 12.80%. Found: C, 58.67%; H, 4.28%; N, 12.92%. FT-IR (KBr) cm^-1 ^1589 C=N (azomethine) stretch band, 3322 N-H stretch band.

*1-Methylindole-3-carboxaldehyde (3-bromophenyl)hydrazone* (**1o**). Yield 49%, m.p. 165–166 ºC; ^1^H- NMR: δ 3.81 (3H,s), 6.81 (1H, d), 6.99 (1H, d), 7.16-7.28 (4H, m), 7.49 (1H, d), 7.67 (1H, s), 8.10 (1H, s, azomethine-CH), 8.18 (1H, d), 10.07 (1H, s, hydrazine-NH); ^13^C-NMR: δ 33.35, 110.83, 111.07, 112.15, 114.14, 120.41, 121.06, 122.20, 123.13, 125.24, 131.75, 132.44, 136.55, 138.17, 148.29 (azomethine-C); ESI MS m/z 328 (M^+^, %100), 330 (M+2, %100), 331 (M+3); Anal. Calcd. for C_16_H_14_N_3_Br: C, 58.55%; H, 4.41%; N, 12.80%. Found: C, 58.78%; H, 4.42%; N, 12.75%. FT-IR (KBr) cm^-1 ^1593 (C=N, azomethine stretch), 3436 (N-H stretch).

*1-Methylindole-3-carboxaldehyde (4-bromophenyl)hydrazone* (**1p**). Yield 68.6%, m.p. 195–196 ºC; ^1^H-NMR: δ 3.80 (3H,s), 7.00 (2H, d), 7.24 (2H, m), 7.36 (2H, d), 7.65 (1H, s), 8.10 (1H, s, azomethine-CH), 8.22 (1H, d,), 10.18 (1H, s, hydrazine-NH); ^13^C-NMR: δ 33.34,108.71, 110.76, 112.30, 113.93, 120.99, 122.41, 123.09, 125.20, 132.38, 136.03, 138.15, 146.02 (azomethine-C); ESI MS m/z 328 (M^+^, %100), 330 (M+2, %100), 331 (M+3); Anal. Calcd. for C_16_H_14_N_3_Br: C, 58.55%; H, 4.30%; N, 12.80%. Found: C, 58.16%; H, 4.53%; N, 12.51%. FT-IR (KBr) cm^-1 ^1589 (C=N, azomethine stretch), 3440 (N-H stretch).

### 3.4. General procedure for the synthesis of compounds ***1r–s***

A solution of 1-methyl-1*H*-indole-3-carboxaldehyde (0.5 mmol) and anisic acid hydrazide or izonicotinic acid hydrazide (0.5 mmol) in EtOH (50 mL) was heated for 2.5 h on a hot water bath. On cooling, the precipitate was collected washed with cold EtOH to give **1r–1s** with 15 to 25% yield.

*N-(4-methoxybenzoyl)-N’-(1-methylindolyl-3-methylene)-hydrazine* (**1s**). Yield 15.3%, m.p. 254–255 ºC; ^1^H-NMR: δ 3.835 and 3.838 (6H, brs, NCH_3 _and OCH_3_), 7.04 (2H, d), 7.20 (1H, t), 7.27 (1H, t), 7.50 (1H, d), 7.80 (1H, s), 7.92 (1H, d,) 8.31 (1H d), 8.58 (1H, s, azomethine-CH), 11.39 (1H, brs, hydrazine-NH); ^13^C-NMR: δ 33.47, 56.09, 110.87, 111.51, 114.33, 121.27, 122.83, 123.35, 125.44, 126.74, 129.98, 134.40, 138.23, 144.53 (azomethine-C), 162.39 (C=O), 162.63, 167.63; ESI MS m/z 308 (M+1), 330 (M+Na, 100%); Anal. Calcd. for C_18_H_17_N_3_O_2_Br: C, 70.34%; H, 5.75%; N, 13.67%. Found: C, 70.07%; H, 5.59%; N, 13.63%. FT-IR (KBr) cm^-1 ^1608 (C=N, azomethine stretch), 3194 (NH-CO stretch).

### 3.5. General procedure for the synthesis of compound ***1t***

A solution of 1-methyl-1*H*-indole-3-carboxaldehyde (2 mmol) and hydrazine hydrate (1 mmol) in EtOH (25 mL) was heated for 4 h on a hot water bath. On cooling, the precipitate was collected washed with cold EtOH to give *N,N^’^-bis-(1-methylindole-3-ylmethylene)hydrazine* (**1t**) in 34% yield. m.p. 233–234 ºC; ^1^H-NMR: δ 3.85 (6H, s), 7.27 (4H, tt), 7.52 (2H, d), 7.89 (2H, s), 8.36 (2H, d), 8.87 (2H, s, azomethine-CH); ^13^C-NMR: δ 33.62, 1MS mass m/z 315 (M+1, %100), 316 (M+2); Anal. Calcd. for C_20_H_18_N_4_: C, 76.40%; H, 5.77%; N, 17.82%. Found: C, 76.41%; H, 5.47%; N, 17.75%. FT-IR (KBr) cm^-1 ^1571 (C=N, azomethine stretch). 

### 3.6. In Vitro Antioxidant Activities

#### 3.6.1. Erytrocyte Isolation

Blood samples were collected into heparinized tubes. The samples were centrifuged for 15 min at 3,000 rpm at +4 ºC. After removing the plasma and the buffy coat, RBCs were washed in equal volume of cold NaCl (0.155 mol/L) for three times. Following the third saline wash, supernatants were removed and packed RBCs were obtained.

#### 3.6.2. Estimation of Reactive Oxygen Species by DCFH-DA

For estimation of ROS inside erythrocytes DCFH-DA was used as a probe. In cellular systems non fluorescent probe DCFH-DA readily crosses the cell membrane and undergoes hydrolysis by intracellular estrases to nonfluorescent 2',7'-dichlorofluorescin (DCFH). DCFH is then rapidly oxidized in the presence of reactive oxygen species to highly fluorescent 2′,7′- dichlorofluorescein (DCF) [28]. In our study, 1% erythrocyte suspension was incubated at 37 ºC in phosphate buffer (50 mM, pH 7.4) and DCFH-DA (20 μM) for an hour. At the end of incubation period, the erythrocyte suspension was washed with PBS three times, resuspended in PBS, pipetted onto a black 96-well plate and various concentrations of melatonin or its derivates were added into the wells. The production of fluorescent DCF was measured using a multiplate spectrofluorometer (excitation wavelength = 488 nm, emission wavelength = 525 nm) [29].

#### 3.6.3. Measurement of H2O2-induced lipid peroxidation levels

Lipid peroxidation was assesed by the determination of malondialdehyde (MDA) levels using the method of Gutteridge *et al.* [30] and Quinlan *et al.* [31] based on the reaction of MDA with thiobarbituric acid (TBA) at 95 ºC. In the TBA test reaction, MDA and TBA react to form a pink adduct with an absorption maximum at 532 nm. The reaction was performed at pH 2–3 at 95 ºC for 15 min. Erythrocytes were resuspended in phosphate buffer (50 mM, pH 7.4) with 7.8 mM azide and different concentrations of melatonin or its derivates were added. The samples were preincubated for 30 min at 37 ºC, added 5 mM H_2_O_2_ and incubated for 2 h at 37 ºC. The sample was mixed with 28% (w/v) trichloroacetic acid to precipitate the protein. The precipitate was pelleted by centrifigation and an aliquot of supernatant was reacted with 1% (w/v) TBA in a boiling water-bath for 15 min. After cooling, the absorbance was read at 532 nm. 

#### 3.6.4. Determination of Erythrocyte Hemolysis

Erythrocytes were resuspended in phosphate saline (PBS: 150 mM NaCl, 1 mM Na_2_PO_4_ and 1.9 mM NaH_2_PO_4_, pH 7.4) at a 20% (v/v) suspension. Erythrocytes at 5% (v/v) suspension in PBS were incubated with 75 mM AAPH in the presence of different concentrations of melatonin or its derivates for 5 h at 37 ºC in a gently shaking water bath. Aliquots were taken out from this mixture at appropriate intervals and centrifuged 2000 rpm for 5 min to obtain supernatant. The absorbance of the supernatant was determined spectrophotometrically at 540 nm [32]. The percentage of hemolysis at different incubation intervals was compared with that of complete hemolysis. For reference, erythrocytes were treated with distilled water and the absorbance for the hemolysate at 540 nm was used as 100% hemolysis. Every experiment was repeated three times and the lag time of hemolysis (t_lag_) was determined. 

#### 3.6.5. Statistical analysis

Unpaired t test was performed to evaluate the significance of the differences between groups. p < 0.05 was accepted as significant.

## 4. Conclusions

In general all the synthesized indole derivatives were found to have potent antioxidant activity, even higher than MLT itself, according to the results of three *in vitro* antioxidant experiments revealing differences in their relative potencies probably related to electronic distribution. No significant antioxidant activity was observed in two compounds **1r**, **1s**. These compounds were the isonicotinic (**1r**) and anisic acid (**1s**) hydrazides of indole 3-aldehydes and they have no halogen atoms in their structure that makes them different from the rest of the synthesized compounds. Structural investigation of the rest of the active compounds showed that having o- and m- halogenated aromatic side chain increase the antioxidant activity (such as compounds **1b**, **1c**, **1m**, **1k** and **1l**). These are the most promising compounds that should be kept in mind for designing new MLT-based indole derivatives for our ongoing study. These results suggest a new approach for the *in vitro* antioxidant activity properties and structure activity relationships of 1,3-disubstituted indole rings. Lack of a methoxy group in the 5 position did not affect the antioxidant capacity of the new indole derivatives. In fact, the *in vitro* assays showed that lack of a methoxy group, introduction of a methyl group at the nitrogen in the indole ring and a halogenated aromatic side chain resulted in much more active compounds than MLT itself. This may be due to increased stability of the indole ring and delocalization of the electrons to help to scavenge free radicals by forming stable indolyl cation radicals. 
